# Is physical activity related to a reduction in the severity of borderline personality disorder through less severe insomnia disorder?

**DOI:** 10.1192/j.eurpsy.2023.2129

**Published:** 2023-07-19

**Authors:** V. Krieger, S. St-Amour, P. Bernard, L. Cailhol

**Affiliations:** 1 Department of Psychology, University of Fribourg, Fribourg, Switzerland; 2 Department of Physical Activity Sciences, University of Quebec at Montreal; 3 Department of Psychiatry and Addictology, University of Montreal, Montreal, Canada

## Abstract

**Introduction:**

Borderline personality disorder (BPD) is associated with severe suffering and insomnia disorder (ID) (Fertuck et al., 2016; Galbiati et al., 2020).

**Objectives:**

The aim was to investigate the negative association between self-reported physical activity (PA) and the severity of BPD with ID acting as a mediator (St-Amour et al., 2021).

**Methods:**

The role of ID within the association of PA with BPD was tested using mediation analysis with the statistical program R 4.3 (N = 120; RStudio Team, 2020).

**Results:**

**Table 1:** Mediation analysis results

	β	se	t	p	LLCI	ULCI
Effect a	0.07	0.05	1.46	0.15	-0.03	0.17
Effect b	0.41	0.09	4.60	< 0.001	0.23	0.59
Effect c	0.11	0.05	2.16	0.03	0.01	0.21
Effect c’	0.08	0.05	1.70	0.09	-0.01	0.17

*Note*: β = beta coefficients; se = standard error; t = t-value; p = p-value; LLCI = lower limit confidence interval; ULCI = upper limit confidence interval. Effect c’: The association within the mediation analysis is not significant (β = 0.08, se = 0.05, p = 0.09). Effect a: PA is not significantly associated with ID (β = 0.07, se = 0.05, p = 0.15). Effect b and c: ID (β = 0.41, se = 0.09, p < 0.001) and PA (β = 0.11, se = 0.05, p = 0.03) are significantly associated with the severity of BPD.

**Image:**

**Figure:**
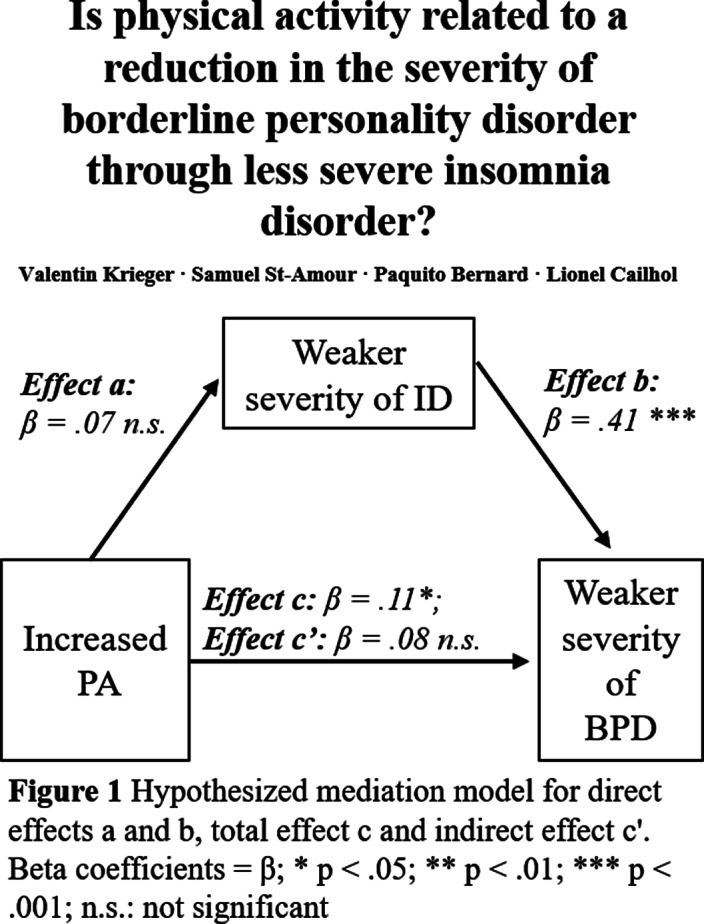

**Conclusions:**

Accordingly, ID does not appear to affect the association of PA and BPD severity whereas fewer PA and severe ID can nonetheless have a positive association with the symptoms of BPD in independent ways.

**Disclosure of Interest:**

None Declared

